# Early career interview: Amy Winship

**DOI:** 10.4155/fsoa-2019-0012

**Published:** 2019-05-03

**Authors:** Amy Winship

**Affiliations:** 1 Research Fellow, Monash Biomedicine Discovery Institute, Melbourne, Australia

Amy Winship was one of five finalists for the Future Science Early Career Research Award 2018. Read her interview to find out about her career, hopes for the future and advice to other early career researchers.

## Please tell us about your career history to-date

My research expertise is in female reproduction. During my PhD at the Hudson Institute of Medical Research, Australia, I established an animal model for the life-threatening pregnancy complication pre-eclampsia. In a separate study, I also demonstrated efficacy of a treatment for uterine cancer that may preserve fertility. In 2016, I received a Cancer Council Victoria Postdoctoral Fellowship to continue research on this novel, fertility-preserving therapeutic strategy for treating uterine cancer and was awarded a commendation at the Victorian Premier’s Awards for Health and Medical Research.

I have published my research in top reproductive and interdisciplinary journals on diverse stages of pregnancy, including endometrial preparation for pregnancy, embryo implantation, decidua formation, placental development and pregnancy complications including spontaneous, preterm birth and low birth weight. My broad reproductive expertise therefore makes me uniquely equipped to lead a new project focused on the impacts of anticancer treatments on the uterus and subsequent pregnancy. This research is highly relevant to population health in the context of fertility of female cancer survivors.

My experience in pregnancy and gynecological cancer research drove my interest in developing fertility-preserving techniques for women undergoing cancer treatment. After being recruited to Monash University, Australia, I was awarded an Australian Government National Health and Medical Research Council Early Career Fellowship (2017–2020). In order to develop novel fertility-preserving techniques, I have partnered with Dr. Hutt, a world leader in identifying mechanisms of anti-cancer treatment-induced oocyte DNA damage in the ovary. We have already published two reviews and two original research articles in less than 2 years. The combination of my expertise and conception of this study, together with Dr Hutt’s laboratory, is therefore tailored perfectly to ensure the success of my current research.

## What made you choose a career in your field?

The ability to work as part of a team and contribute to meaningful and impactful science is what drove me to choose a career in medical research. The ability to think creatively and create new knowledge through the rigor of a well-designed study are what drives me to continue my career.

## Describe the main highlights of your career so far

The best thing about my research career has been the relationships I have formed with my mentors and other lab members. These are rewarding relationships that will be long-lasting and help to drive me forward, not only as a better scientist, but as a better person.

## Describe the most difficult challenge you have faced & how you overcame it

My personal challenge in science has been to overcome striving for perfection. Research science is competitive due to the constant search for funding. Therefore, it is important to understand that great scientists do not always get the award of grant – that does not mean that they are not great scientists. Accepting that my best might not always be good enough to secure a grant or award is part of science. That has been a difficult learning curve and I have had to grow a much thicker skin in a short timeframe.

## How do you feel you have impacted your field?

Healthy pregnancy is essential for a healthy start to life. Complications during pregnancy can compromise the immediate, and long-term health and well-being of both the mother and baby. This puts an enormous burden on healthcare systems for years to come. Understanding the causes and improving the treatment of pregnancy disorders is crucial to guarantee the best start to life for all babies. The placenta is the lifeline between the mother and baby and is vital for healthy pregnancy. Abnormal placental development is a leading cause of pregnancy disorders including pregnancy loss, pre-eclampsia and fetal growth restriction.

Placental development, which nourishes a growing baby, and cancer growth and spread have a striking similarity; placental and tumor cells both grow and replicate rapidly. They also invade into the womb and if this invasion is not monitored, it can cause disease.

Uterine cancer is the most common gynecological cancer worldwide and the fourth most common cancer in women, killing approximately 75,000 women worldwide and around 2500 women in Australia annually. Alarmingly, its incidence is rising, particularly in women of reproductive age. There is no screening test and a lack of curative therapies for patients with recurrent or aggressive disease, or young women wanting to preserve fertility.

During my PhD studies, I identified a single molecule called ‘IL-11’, which causes abnormal cell growth and invasion in the womb. I have since demonstrated that elevated levels of this small signalling protein cause both pregnancy complications and uterine cancer. Excitingly, my studies show that blocking IL-11 can promote healthy pregnancy; stop uterine cancer growth and spread; and importantly preserve the future fertility of these women. I highlighted for the first time that abnormal levels of IL-11 cause serious pregnancy complications including pre-eclampsia. I then developed a new mouse model of pre-eclampsia. This is significant, as previous models do not exhibit the full spectrum of disease features, which has hampered therapeutic testing. This model is now being used by my past research group and others around the world as a preclinical model to test urgently needed treatments. I also provided evidence for efficacy of a novel nonchemo-/radio-therapy and non-hormonal treatment for uterine cancer, by blocking IL-11 proteins of interest. As a result, I am a named investor on a patent to develop this into a translational therapy.

## What are your aims for the future?

My goal is to lead a world-class reproductive research team to improve fertility, pregnancy outcomes and health for the future. My current research capitalizes and expands on my expertise in developing mouse models of cancer and reproductive disorders to identify novel mechanisms of uterine damage after cancer therapy. My overall future goal is to exploit these targets to develop therapeutics to prevent damage to the uterus and improve fertility. I will use my primary human cell and tissue culture experience to drive translational studies in women and position myself as an internationally competitive researcher in fertility regulation. I have already secured my salary for the duration of my current research focus and have recruited a PhD student to drive this research. I was recently awarded competitive seed funding from Monash University to establish the embryo transfer models in mice, demonstrating my ability to be competitive for larger external funding applications in the future.

My research is the very first step in uncovering entirely new knowledge about the exact impacts of anti-cancer treatments on the uterus and its ability to foster a healthy pregnancy after treatment has ceased. My short-term goal is to gain much needed knowledge regarding the precise extent of damage that radiotherapy or different chemotherapy drugs, commonly used to treat young cancer patients, can cause to the womb. Only once we understand how anti-cancer treatments damages the uterus, will we be able to design appropriate intervention methods to protect, or restore its normal function. Importantly, my study will assess each key stage of pregnancy. Because I am using donor embryos in recipient mice exposed to cancer therapies, I will know for certain that any differences between the control or treated animals are caused by defects of the uterus, but not the ovary and eggs. Assessing the impact of cancer therapies on each of the key stages of successful pregnancy in my animal model will uncover new information regarding the reasons why cancer survivors are more susceptible to pregnancy complications than women who have not been exposed.

My medium-term goal, which I strive to achieve soon, will be to test whether our findings in an animal model are also relevant to women. To do this, I will test our findings using human uterine tissues samples collected from female cancer survivors undergoing IVF. This will help me achieve my long-term goal, to substantially impact the clinical care of young cancer patients and survivors. This includes ensuring that fertility-preservation counselling becomes standard care for all young patients and that they are aware of the potential impacts of radiotherapy and chemotherapy on the uterus. My long-term goal is to use the findings from this study to develop innovative and much-needed approaches to protect the uterus and fertility in young women being treated for cancer.

## What advice do you have for those hoping to win the award in the future?

My advice would be to apply for the award, even if you do not think you’re competitive. Although I did not win, I did not even think I would be shortlisted after my supervisor told me she had nominated me. You have to be in it to win it!

